# Wearable Sensors for Sleep Monitoring in Free-Living Environments: A Scoping Review on Parkinson’s Disease

**DOI:** 10.3390/bios15040212

**Published:** 2025-03-25

**Authors:** Joana Matos, Beatriz Ramos, Joana Fernandes, Clint Hansen, Walter Maetzler, Nuno Vila-Chã, Luís F. Maia

**Affiliations:** 1 Department of Neurology, Centro Hospitalar Universitário de Santo António, 4099-001 Porto, Portugal; 2 Department of Neurology, Kiel University, 24105 Kiel, Germany; 3 Instituto de Ciências Biomédicas Abel Salazar, Universidade do Porto, 4050-313 Porto, Portugal; 4 i3S—Instituto de Investigação e Inovação em Saúde, Universidade do Porto, 4200-135 Porto, Portugal

**Keywords:** wearables, Parkinson’s disease, sleep–wake disorders, ambulatory, machine learning, sleep, actigraphy

## Abstract

Parkinson’s disease (PD) is a neurodegenerative disorder that affects multiple neural pathways, leading to a broad spectrum of motor and non-motor symptoms. Sleep disorders, such as insomnia and excessive daytime sleepiness, are prevalent among PD patients and significantly impact symptomatology and patients’ quality of life. Wearable technology presents an opportunity to study these interactions in patients’ daily life environments without the limitations of in-clinic sleep studies. Thus, this review aims to explore how wearable technology has been employed or developed for the sleep monitoring of PD patients in free-living environments. A comprehensive search was conducted across PubMed, Scopus, and IEEE Xplore to identify original research articles focusing on wearable sleep technology for the ambulatory monitoring of PD patients. Twenty-six studies fulfilled the inclusion criteria and underwent structured data extraction and quality assessment. Key aspects analysed included subject demographics, extracted sleep parameters, identified sleep disorders, and the application of machine-learning algorithms. Wearable devices could offer a practical solution for long-term sleep monitoring in PD, though further validation is needed. The absence of standardised protocols and the lack of device validation within PD populations remain significant challenges. The evidence gathered in this study remains insufficient to define a standardised protocol for sleep assessment of PD patients in free-living environments.

## 1. Introduction

Parkinson’s disease (PD) is a neurodegenerative disorder typically diagnosed based on the presence of motor symptoms such as bradykinesia, tremor, and rigidity. However, the involvement of several neural pathways within the central and peripheral nervous systems results in a wide range of clinical manifestations. Some non-motor symptoms, such as hyposmia, depression, and sleep disruption, can precede the motor symptoms by several years [1].

Sleep disturbances are amongst the most prevalent features of PD and affect up to 80% of patients five years after diagnosis. These include insomnia, excessive daytime sleepiness (EDS), restless legs syndrome (RLS), and rapid eye movement (REM) sleep behaviour disorder (RBD). With age, the frequency of these symptoms increases, impacting the patient’s daily functioning [2].

Sleep itself appears to impact symptom severity. The loss of slow-wave sleep, associated with disease progression, has been linked to motor and cognitive decline. Conversely, some patients experience improved symptoms after waking up, a phenomenon called sleep benefit that appears to be associated with stable nocturnal sleep [2].

The influence of standard PD medication on sleep has revealed complex results. For example, Levodopa and dopamine agonists, the first line of treatment for PD, have been shown to improve some sleep disturbances like RLS but seem to exacerbate insomnia, EDS, and nighttime hallucinations. These distinct effects appear to be dependent on the dosage and timing of administration [2,3,4]. Sleep disturbances can be compounded by the effect of dopamine agonists on impulse control disorders that might cause sleep deprivation [2].

Although sleep has a significant impact on quality of life, its assessment is still limited. It is dependent on questionnaires and interviews of patients and spouses, which are frequently affected by recall bias, or on polysomnography, which is associated with high cost and time demands and consequently is not easily accessible [3,5].

Wearable technology may provide a solution to some of these limitations. It enables the continuous, non-obtrusive, and objective monitoring of patients in their ecological environments. Wearable devices may also detect subclinical outcomes and facilitate the measurement of sleep-related variables at multiple time points. By increasing the amount of available information, wearables not only provide a more comprehensive picture of PD patients but may also contribute to achieving statistical significance in clinical trials [5].

Based on this information, this scoping review aims to explore how wearable technology has been employed to monitor sleep and diagnose sleep disorders in PD patients in free-living environments, providing an overview of the population, technology, outcomes, and applications across diverse studies.

## 2. Methods

This scoping review follows the recommendations of the Preferred Reporting Items for Systematic Reviews and Meta-Analyses extension for Scoping Reviews (PRISMA-ScR) [6]. The protocol of this review was not registered.

### 2.1. Search Strategy

To identify relevant studies, PubMed, Scopus, and IEEE Xplore were systematically searched. Databases were searched up to 20 August 2024, with no other limit to publication date. Four groups of search terms were created, related to wearable sensors, Parkinson’s disease, sleep, and ambulatory monitoring. When available, Medical Subject Headings (MeSH) terms were used. Terms related to wearable sensors included “Actigraphy”, “Body-worn”, and “Mobile device”. Terms related to sleep included “Sleep Wake Disorders”, “Insomnia", and “REM”. Terms related to ambulatory monitoring included "Free-living”, “ecological” and “unsupervised”. An example of the full search queries is presented in Table 1.

### 2.2. Eligibility Criteria

This review focuses on original peer-reviewed research. Thus, abstracts, editorials, reviews, and protocols were excluded. Works were only included if they were written in English. Studies were included if they incorporated any measure of sleep parameters. These were defined as any parameters that directly describe sleep, such as total sleep time and sleep latency, parameters related to mobility or physiological signals during the night, and any other parameters that were related to specific sleep disorders such as RBD, insomnia, and EDS. Studies that did not use wearable sensors, defined as any non-invasive electronic sensor that can be comfortably worn continuously on the human body, to measure these sleep parameters were also excluded. Studies that did not explicitly include PD patients or that only measured parameters in laboratory, clinical, or other structured environments were not included either. Interventional studies and studies using only animal or in vitro models were also excluded.

### 2.3. Selection Process

Duplicate papers were manually removed by one of the reviewers (JM). Three independent reviewers (JM, BR, and JF) screened the titles and abstracts of all studies. Full-text screening was once again performed by the three reviewers. Studies were included based on majority voting.

### 2.4. Data Extraction

Data for each included study were collected by one reviewer (JM) using a structured form. The study investigators were not contacted to obtain any relevant missing data.

The data retrieved included the following: study metrics such as authors, publication year, journal, and funding; information about the sensing technologies employed, including the sensing modality, name of sensors, number of sensors, sensor position, and sampling frequency; the characteristics of participants, such as the number of PD patients and controls included, sex, age, disease stage, and inclusion and exclusion criteria; the clinical scales and questionnaires employed; the extracted sleep parameters; the correlation of sleep with other disease domains; and machine-learning algorithms developed or used and the achieved metrics. Study goals were divided into three categories: technological validation if the study introduced a novel device; algorithmic validation if the study validated a new data-processing method using commercial or previously introduced hardware; and clinical research if both the hardware and algorithms were commercial or had been previously introduced, or if the study focused on the characterisation of patients.

Characteristics such as extracted parameters and study goals were only retrieved if they pertained to the evaluation using wearable sensors in ambulatory monitoring. Data obtained from non-wearable sensors or in clinic were only recorded if they were used for comparison.

In the cases where monitoring duration was not explicit, the average of number of nights per participant was calculated and is here presented as an estimate. If not stated, the country of recruitment of the participants was assumed to be the country of the authors.

### 2.5. Quality Assessment

A quality assessment was performed following data extraction to assess the risk of bias in the included studies. This assessment was based on the work of Downs and Black [7]. As in the work of Milane et al., this list was shortened to exclude the questions that do not apply to non-interventional studies [8]. The items cover reporting clarity and transparency, external validity, and intervention and subject selection bias.

## 3. Results

### 3.1. Study Selection

**Figure 1:**
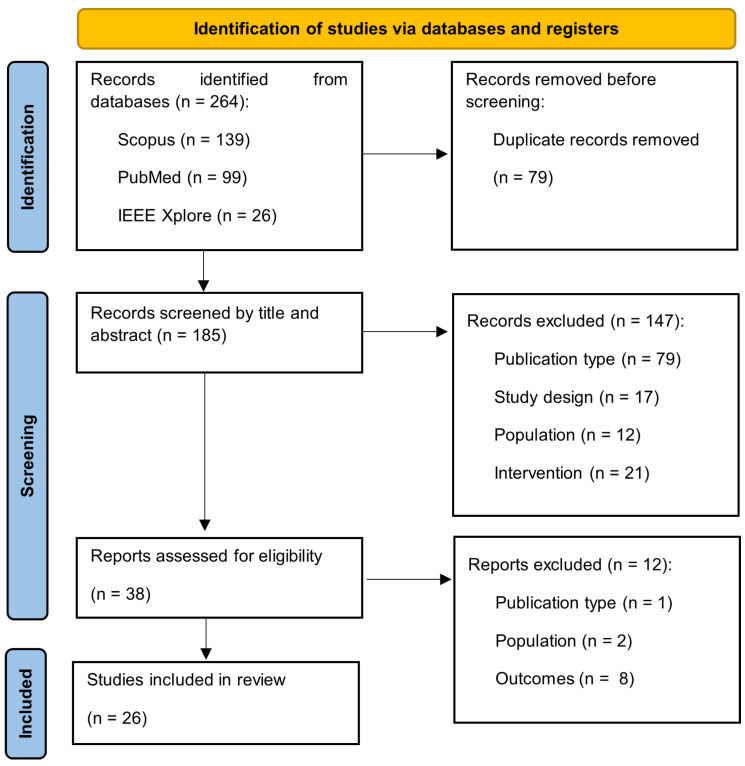
Inclusion process.

The selection process is depicted in Figure 1. The database search identified a total of 264 papers: 139 from Scopus, 26 from IEEE Xplore, and 99 from PubMed. The removal of duplicates (*n* = 79) resulted in 185 papers. These papers were screened based on titles and abstracts, which resulted in 38 papers being included in the full-text screening.

Studies were excluded based on pre-established criteria, including population, study environment, outcome measures, and intervention type. Following this selection process, 26 articles [9,10,11,12,13,14,15,16,17,18,19,20,21,22,23,24,25,26,27,28,29,30,31,32,33,34] met the inclusion criteria and were incorporated into this review.

### 3.2. Study Characteristics

Table 2 summarises the main characteristics of the included studies. These include the publication year, main goal of the study, sensor modalities and positioning, monitoring duration, and key takeaways. The studies were divided into three main goals: 11% focused on validating new technologies, 27% on validating algorithms, and 62% on clinical research. The median (IQR) of the monitoring duration was 3 (1.8–7).

The list of the sensors used in each study is available in the Supplementary Materials.

### 3.3. Quality Assessment

Table 3 includes the scores of the quality assessment for each study; 96% of studies clearly described their primary objective, and 69% explicitly presented the main outcomes to be measured. Meanwhile, 77% clearly described the characteristics of included and excluded patients. Among the applicable studies, 76% listed the principal confounders between groups, while 10% listed them partially. Additionally, 65% adequately adjusted for confounding, 41% ensured recruitment over the same population, and 19% recruited them over the same time period. While 96% presented their major findings clearly, 100% presented these data with estimates of their random variability. Of the reviewed studies, 15% made it clear that the persons asked to participate were representative of the population, and no studies (0%) demonstrated that those willing to participate were representative of the broader population initially approached for recruitment. All studies (100%) employed appropriate statistical tests and valid outcome measures. However, only 67% made explicit the probability values of the main outcomes. The one study that included follow-up adjusted for the length of follow-up but failed to explicitly describe the characteristics of patients lost to follow-up or take them into account in the analysis.

### 3.4. Participant Characteristics

**Figure 2:**
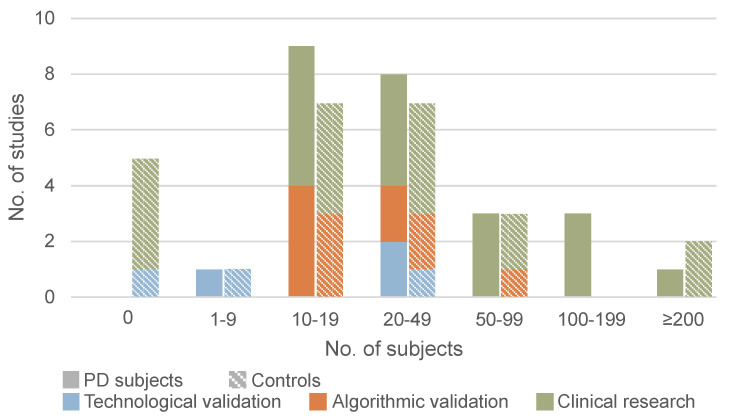
Number of PD patients and controls in the included studies.

The information regarding the participants included in each study are presented in Table 4. The median (IQR) number of PD patients included was 21 (17–51), and the median number of controls was 19 (9–30). Five studies did not include any controls. Figure 2 illustrates the distribution of the number of participants according to the primary objective of each study.

**Figure 3:**
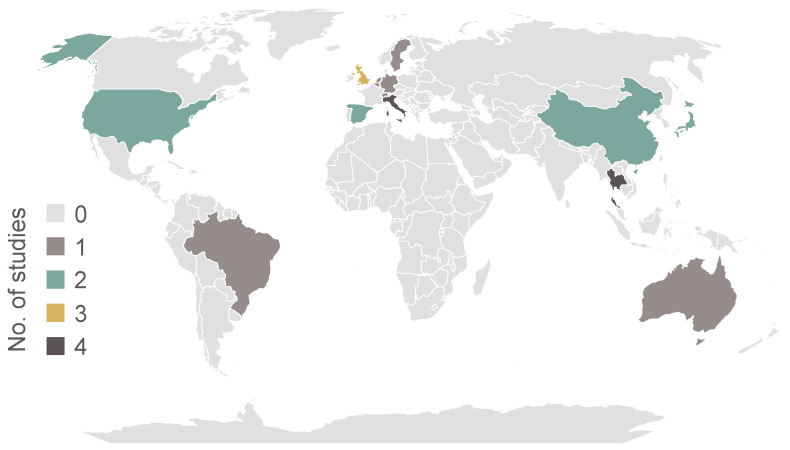
Geographical distribution of PD patients included in the studies.

Across all studies, 29% of PD patients and 52% of controls were female. The mean age of PD patients ranged between 64.5 and 74.88 years, while controls’ mean age age ranged between 38 and 71.9 years. The mean minimum H&Y stage was 1.2 ± 0.4, and the mean maximum H&Y stage was 3.3 ± 0.7. Only one study included patients with H&Y stage 5. The geographical distribution of the countries from which PD patients were recruited is shown in Figure 3.

### 3.5. Sensor Type and Position

**Figure 4:**
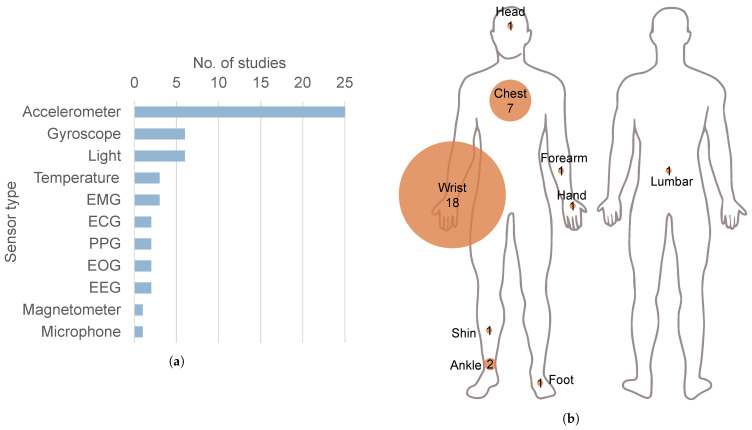
(**a**) Modalities of sensors used. EMG: electromyography. ECG: electrocardiography. PPG: photoplethysmography. EOG: electrooculography. EEG: electroencephalography. (**b**) Position of the accelerometers on the participants.

As can be seen in Figure 4a, all but one of the studies used an accelerometer. Gyroscopes and light sensors were used in six studies. Electromyography (EMG) was used to measure muscle activity in the mentalis muscle [15], in three frontopolar electrodes [22], and in the chest, shin, forearm, and back of the hand [26]. Electroencephalography (EEG) was recorded through the same three frontopolar electrodes [22] and four electrodes on the forehead [15]. Other types of electrography included electrocardiography (ECG) [23,27] and electrooculography (EOG) [15,22]. Magnetometers [21] and microphones [22] were each used in one study.

**Figure 5:**
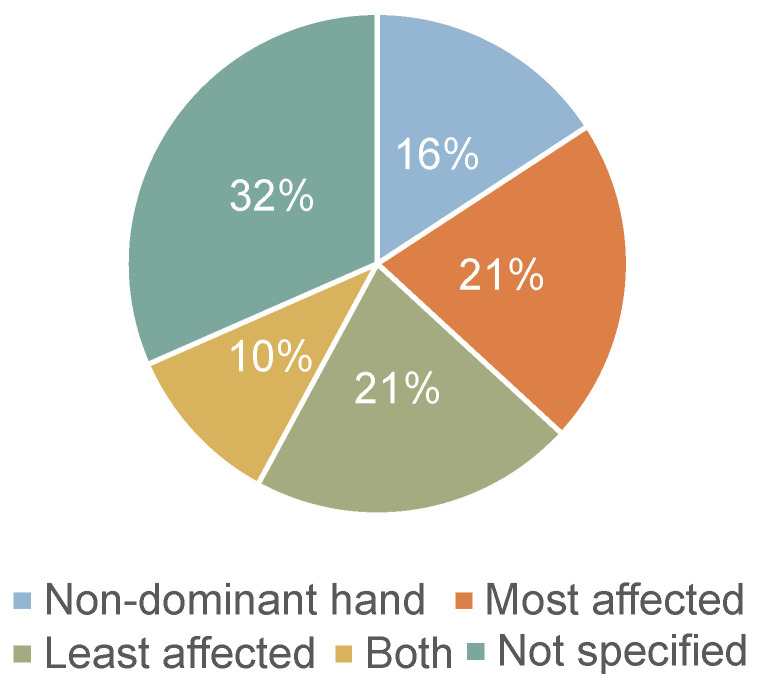
Wrist side of accelerometers in PD patients.

Figure 4b shows where the accelerometers were placed on the body. Eighteen of the studies used at least one accelerometer on the wrist. Of these, as can be seen in Figure 5, three studies placed them on the non-dominant side [17,25,34], four on the side most affected by the disease [26,30,31,33], four on the side least affected [9,14,27,28], and two on both wrists [11,20]. While, in the study by Raschellà et al. [30], the main outcomes were derived from using just one accelerometer, a subset of these patients with asymmetric motor deficits was used to test sensor placement. The best performance of the RBD classifier resulted from using the accelerometer on the most affected side and using sensors on both wrists did not improve classification performance.

### 3.6. Correlation with Other Disease Domains

**Figure 6:**
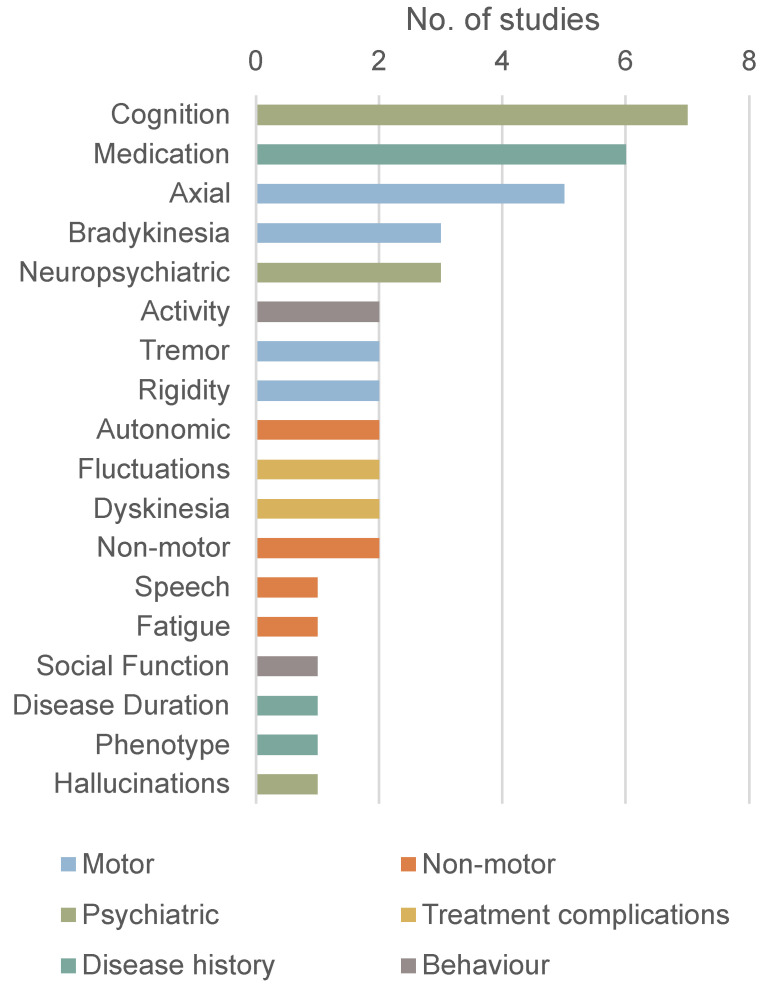
No. of studies that correlated sleep with other disease domains.

Multiple studies examined the relationship between sleep deficits and other disease domains that affect PD patients, as shown in Figure 6. Wu et al. found no association between actigraphy sleep parameters and subjective levels of anxiety, cognition, fatigue, or social function [10]. Kotschet et al. found that differences in quality of life and cognitive function were not statistically significant in patients with more daytime sleepiness [31]. In the work of Prudon et al., cognitive impairment (MoCA and MMSE) was not correlated with any measure of sleep disturbance [13]. However, in the study by Schalkamp et al., sleep measures correlated with cognitive function, and Whitehead et al. found significant correlations between the rest–activity rhythm and cognitive function [23,28]. In the research of Höglund et al., anxiety was associated with daytime sleepiness in patients classified as non-fluctuators [33]. In the study by Whitehead et al., hallucinating PD patients had higher circadian rhythm disruption when compared with non-hallucinating PD patients. Significant correlations were also found between the rest–activity rhythm and complications of therapy measured using UPDRS-IV [28]. In the work of Schalkamp et al., sleep measures correlated with activity levels, UPDRS-IV, and autonomic function [23]. Non-motor symptoms’ severity was significantly correlated with nocturnal movement in the study by Mirelman et al. [12]. In the research by Madrid-Navarro et al. (2018), the day/night activity ratio was linked with chronodisruption scores of wrist temperature, sleep probability (based on temperature, motor activity, and body position), and nocturnal time in movement but did not correlate with UPDRS [14].

In Mirelman et al., nocturnal movement was significantly correlated with Levodopa equivalent daily dose (LEDD) [12]. Gnarra et al. found no correlation between LEDD and sleep positions [22]. No significant association between LEDD and rest activity or HRV was observed in Niwa et al. [27]. There was no significant association between medication dose and daytime sleepiness [31].

In the work of Mirelman et al., nocturnal movement was significantly correlated with motor severity (measured via MDS-UPDRS-III), rigidity, bradykinesia, and postural instability gait difficulty [12]. Kotschet et al. and Höglund et al. found associations between daytime sleepiness and bradykinesia and dyskinesia [31,33]. In patients classified as fluctuators, there was also an association between daytime sleepiness and the fluctuation score [33]. However, differences in disease duration and motor function were not statistically significant [31]. In the research by Gnarra et al., REM sleep in the supine position correlated with disease duration and UPDRS-III, while slow wave sleep correlated with the H&Y stage [22]. In the study by Niwa et al., rest activity and HRV were associated with UPDRS and UPDRS-III [27]. Whitehead et al. found significant correlations between the rest–activity rhythm and motor function (UPDRS-III) [28]. In the work of Prudon et al., disease severity was associated with increased periodic limb movements [13]. Nocturnal movements were altered even in early-stage patients [12].

### 3.7. Sleep Parameters

**Figure 7:**
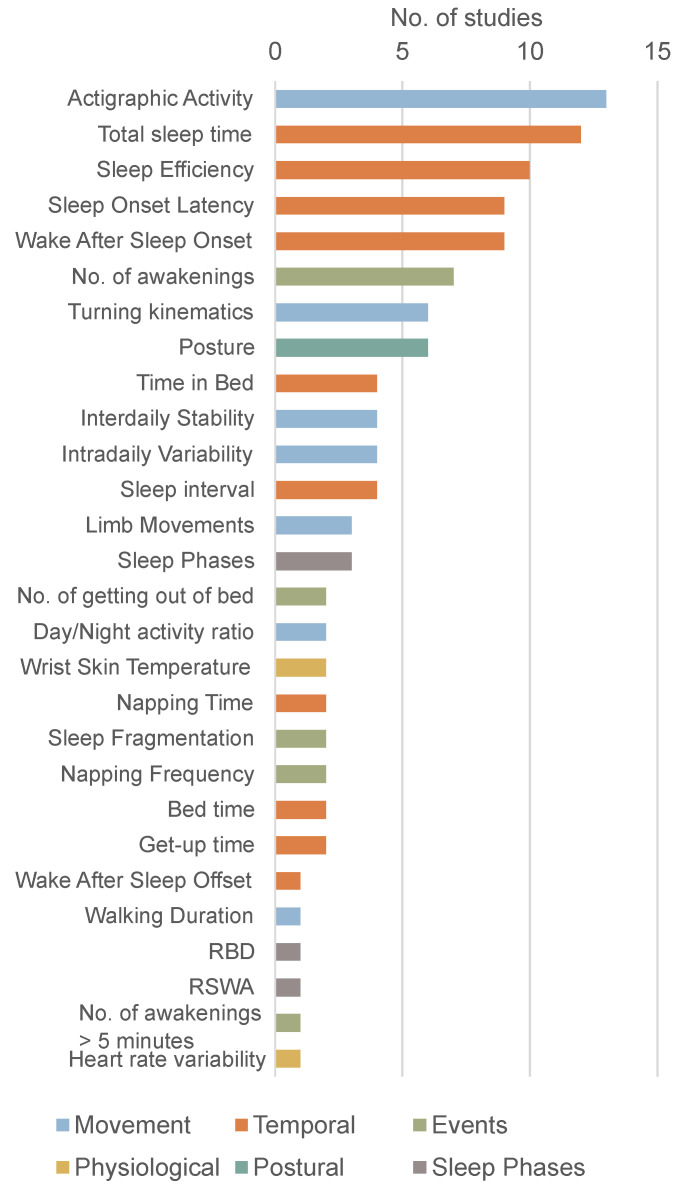
Parameters extracted from the sensors.

As shown in Figure 7, wearable sensors allow for the extraction of a wide range of parameters. This variety is visible not only in the number of parameters but also in the definitions adopted in the studies.

#### 3.7.1. Temporal Parameters

Some of the parameters extracted in these studies are temporal descriptors of sleep, such as total sleep time, sleep efficiency, and sleep onset latency. Bedtime and get-up time are defined based on activity levels, visible light, and skin temperature [9]. Time in bed is the difference between bedtime and get-up time [9] or between lights-off and lights-on [21]. Sleep onset latency (SOL) is the time between bedtime and the first period marked as sleep (sleep onset) [9,25] or the time to the start of the first 20-min block with more than 19 min of sleep [27] and was not significantly different between PD patients and controls [9,27]. The sleep interval is defined as the time between sleep onset and offset [9]. The duration of sleep was considered equivalent in the scope of this review and was defined as the total period while in bed, excluding the first and last 5 min [29]. There were no significant differences in sleep interval [9,16,20,29]. Total sleep time (TST) was defined as the time asleep during the sleep interval [9], simply the total hours of sleep [21] or the time between bedtime and rising time multiplied by the sleep efficiency [25]. TST was higher in controls [9,14,25,32] or not significantly different [11,15,24]. Sleep efficiency (SE) is the percentage of time asleep while in bed [9,15,21,25,27]. SE was higher in controls [9,25,27] or not significantly different [24]. Wake after sleep onset (WASO) was defined as time marked as awake between sleep onset and offset [9,15,25,27]. WASO was consistently higher in PD patients [9,25,27]. Napping time is the time asleep outside the main sleep period [9]. Similarly, % sleep in the out-of-bed period was here considered equivalent to napping time [27]. Both metrics were higher in PD patients. Wake after sleep offset was not explicitly defined [18].

#### 3.7.2. Movement Parameters

Another group of parameters describes nocturnal movement and its relationship to daytime activity. Actigraphic activity was summarised using different measures based on the mean value and central hour of the 5 and 10 consecutive hours with the highest and the lowest values (L5V, L5T, M10V, etc.) [9,14,19,28]. Several studies extracted mean acceleration or activity per time period [9,27,28,34]. Although not always specified, activity is typically measured using activity counts, which are the peak intensity of movement in each time period in arbitrary units [28]. In the work of Madrid-Navarro et al. (2019), actigraphic activity was also measured using the time during the sleep interval in which movement is detected [9]. In the study by Qian et al., activity intensity was defined through the zero crossing mode and the proportional integral mode [17]. In the research by Giganti et al., the total average of motor activity and the average of motor activity in active periods were analysed but not explicitly defined [24]. Boroojerdi et al. extracted multiple time- and frequency-domain features from the accelerometers, such as signal entropy, the root mean square value, spectral entropy, and bandwidth [26]. Raschellà et al. defined the activity rate as the percentage of activity, measured through the magnitude of the acceleration, above a threshold within a sliding window and then extracted several metrics such as the mean, skewness, and kurtosis [30]. Kotschet et al. utilised a device that directly outputs a bradykinesia score. Episodes of at least 2 min with a bradykinesia score below a predefined threshold were used to define episodes of immobility [31,33]. The activity index and the average motility were used in the work of Rechichi et al. [21]. The activity index correlates to physical activity intensity and energy expenditure [35]. The average motility is the average of the activity index over a 2-min window.

In the work of Niwa et al., a metric called a rhythm pattern was introduced as the relation between the activity in the out-of-bed period and the activity in the in-bed period [27]. This metric was higher in controls than in PD patients. In the work of Whitehead et al., the relative amplitude was introduced as a non-parametric descriptor of activity and was calculated as the difference in activity between the most and least active hours over their sum [28].

Interdaily stability (IS) is an indicator of similarity between the activity patterns of different days, and it is related to synchronisation with the 24-hour day-night cycle. Intradaily variability (IV) is a measure of rhythm fragmentation, as it quantifies the frequency and intensity of the transitions between rest and activity. Both metrics are used to characterise the circadian rhythm [9,14,19,28]. IV was significantly higher in PD patients [14,28]. IS was higher in controls [14] or not significantly different [28]. Gonçalves et al. used different sampling frequencies to calculate these metrics [19].

Multiple studies analysed turning movements by measuring the duration, frequency, angular displacement, velocity, acceleration, and torque of these movements [11,12,16,29]. Some studies defined turning movements as a series of trunk movements of at least 15° between two static positions that were held for at least 5 min [11,20,29]. Rechichi et al. defined turning events as the change between sleeping positions both maintained for 2 min [21]. Mirelman et al. defined turning as a change between two static positions with a minimum of 10° [12].

Limb movements were defined as changes of at least 15° and quantified by the number of occurrences per night [11,20]. There were significantly more upper-limb movements in PD patients than in controls [20]. In the work of Prudon et al., limb movements were quantified by the periodic limb movement index, but the detection of limb movements was not specified [13].

Nocturnal walking duration was used in the study by Mirelman et al. to define nocturnal rest interruptions. There was no significant difference between PD patients and controls [12].

#### 3.7.3. Nighttime Events

Multiple studies looked at the frequency of nighttime events such as awakenings [9,10,15,16,18,23,27]. Awakenings were defined as intervals of at least 30 s [9] or simply as blocks of contiguous waking epochs. These were more frequent for PD patients [27] or not significantly different [9,16]. In the work of Mikulec et al., an additional metric of the number of awakenings longer than 5 min was also used [18]. Similarly, getting-out-of-bed activities were identified through rapid rises in acceleration in the x-axis of movements of more than 45°. These were consistently more frequent in PD patients [20,29]. In the study by Mikulec et al., sleep fragmentation was used but not defined [18]. Obayashi et al. defined the fragmentation index (FI) as the percentage of the number of 1 min epochs scored as immobile of the total number of epochs scored as immobile during time in bed. FI was higher in PD patients than in controls [25]. Napping frequency was defined as the number of episodes of sleep outside the main sleep period [9] or the number of blocks of contiguous sleep epochs out of bed [27]. This was consistently higher in PD patients [9,27].

#### 3.7.4. Postural Parameters

Multiple works looked at the participants’ posture during the night [11,12,17,21,22,26]. 3 studies classified sleep positions as supine, prone, left lateral and right lateral based on the alignment of the axes of an axial sensor located in the chest [11,21,26]. Rechichi et al. also quantified the reclining angle in bed and the time spent standing or sitting during the night [21]. Gnarra et al. analysed the head position as supine, lateral right and left, prone right and left, and upright [22]. Qian et al. classified each period as lying or upright posture [17]. Mirelman et al. distinguished between upright and lying based on a threshold of the vertical acceleration. Body position was identified as back, belly, right side, and left side based on the acceleration on the mediolateral and anteroposterior axes [12].

#### 3.7.5. Sleep Architecture and Physiological Parameters

In Oz et al. and Gnarra et al., the data from the wearable systems were scored into the different sleep stages (Wake, N1, N2, N3, and REM) [15,22].

Some studies tracked physiological signals throughout the night. These included wrist temperature, to represent autonomic balance at the skin vessel level [9,14], and heart rate variability (HRV) [27]. Several components of HRV were altered in PD patients when compared to controls [27].

### 3.8. Sleep Disorders

In addition to looking at how sleep metrics are altered in PD patients, some works tried to study specific sleep disorders, as summarised in Table 5.

Four studies looked specifically at nocturnal hypokinesia [11,16,20,29]. Across these studies, PD patients had significantly fewer turns in bed, lower turning speed, acceleration, and torque, and a lower amplitude of turning than controls. This difference was more significant during the second half of the night [11]. Some of these metrics correlated with the nocturnal akinesia dystonia and cramp score (NADCS) [20]. Mirelman et al. achieved similar results when comparing the number, amplitude, and velocity of turns between HC and PD patients. When comparing between different PD stages, the number and velocity of turns decreased with disease severity, while duration increased. Turning duration and velocity were already altered in patients less than 1 year from diagnosis [12].

The number of episodes of getting out of bed was higher in PD patients than their spouses across all studies. However, in the work of Sringean et al. (2017), this difference was only statistically significant during the second half of the night [11]. These episodes were associated with nocturia in all studies [11,20,29].

Prudon et al. found an association between periodic limb movements (PLMI) and disease severity. However, there was no association between increased PLMI and symptomatic restless legs syndrome. The prevalence of sleep-disordered breathing and periodic limb movements of sleep were not increased when compared to population norms [13].

Oz et al. used a wearable device capable of detecting sleep stages. Based on this, they developed a method for detecting RBD. Abnormal REM sleep was identified as REM sleep with increased muscle tone in the EMG channels on the mentalis muscle. RSWA was defined as abnormal REM sleep accompanied by movement artefacts in the EMG data. Comparing the at-home RSWA results with video-polysomnography (vPSG) RBD diagnosis achieved 92.0% accuracy, 100.0% sensitivity, and 89.47% specificity [15]. Raschellà et al. developed an algorithm capable of detecting RBD in PD patients and patients with insomnia based on actigraphic features. The results were compared with vPSG and clinical history and achieved 100% accuracy [30]. Schalkamp et al. found no association between the chosen digital metrics and the RBDSQ, and the implemented model was unable to predict this clinical score. Similarly, no sleep digital metrics correlated with extreme daytime sleepiness (EDS), measured using the Epworth Sleepiness Scale (ESS) [23].

Kotschet et al. divided PD patients into two groups based on ESS scores and showed that the group with the higher score had significantly more periods of immobility measured via actigraphy. Periods of immobility also had 85.2% concordance with sleep periods detected via ambulatory daytime PSG, suggesting that immobility can be used as a metric of daytime sleep. There was no relation between LEDD and immobility. However, there was an increase in sleepiness in 53% of participants after medication intake [31]. Conversely, Höglund et al. found no correlation between periods of immobility and daytime sleepiness, measured using the Karolinska Sleep Scale (KSS) [33].

### 3.9. Machine Learning

As machine-learning (ML) techniques develop, they are increasingly being explored in healthcare to enhance diagnoses, inform decision-making, predict prognoses, and optimise treatment planning. Of the included studies, seven explored the use of machine-learning methods to further analyse sleep in PD patients.

Two studies developed wake/sleep classifiers. In the work of Qian et al., maximum likelihood estimation was used to develop a linear regression model that translates activity intensity into a preliminary wake/sleep state for each 30s interval. The final wake/sleep state was determined based on the combination of the preliminary result and the person’s posture. The model was optimised based on the accuracy of the classification. Ground truth was obtained from video recordings. The model was tested on patients with deep brain stimulation (DBS) off and on and with sensors on the chest and the wrist. Accuracy and sensitivity were higher for the chest sensor, achieving, respectively, 82.74% and 82.68% for DBS off and 85.78% and 84.21% for DBS on. Specificity was 82.28% for DBS off and 82.08% for DBS on [17]. Madrid-Navarro et al. (2019) used a trademarked algorithm to determine the wake/sleep state in each 30s interval. This classification was then used to calculate the other sleep parameters used in the study [9].

Madrid-Navarro et al. (2018) developed a model that classifies participants as PD patients or controls. Parameters related to activity and sleep quality were selected based on entropy reduction. Using a ratio of acceleration and time in movement, all persons were correctly classified [14]. Similarly, Mikulec et al. developed a method to identify prodromal typical and atypical Parkinsonian syndromes using actigraphic and diary data. The method uses the XGBoost classifier trained on diagnoses made by neurology experts. Each person was classified nightly. Majority voting for all nights was used to determine subject-by-subject classification. The model achieved 95.1% accuracy, 100.0% sensitivity, 91.6% specificity, and 0.94 F1 score. The actigraphy features were determined to be more important to the model than the sleep diary features [18].

Rechichi et al. developed two models with distinct goals. A classifier distinguished between healthy controls and PD patients, and another classifier distinguished between good and bad sleep quality (based on the sPSQI). Three methods were tested for each model: support vector machine (SVM), K-nearest neighbours, and XGBoost. The F1 score was used to optimise the models’ robustness. Leave-one-subject-out cross-validation was used for model evaluation. The performance of the different methods was compared using accuracy, recall, and F1 score. SVM achieved the best performance in distinguishing between HC and PD patients, achieving 96.2% accuracy, 95.0% recall, and a 93.4% F1 score. XGBoost was the best method for distinguishing between good and bad sleep quality, achieving 85.7% accuracy, 78.6% recall, and an 82.5% F1 score [21].

Schalkamp et al. used linear regression models to predict clinical measures using the weekly average of the digital data. Diagnosis age, time since diagnosis, and sex were used as covariates. Models were fitted using nested five-fold cross-validation and evaluated based on the R2 score. These models were unable to predict the scores of the clinical scales on an individual level, with most models having an R2 score less than zero [23].

Raschellà et al. tested multiple methods to distinguish between patients with and without RBD: linear discriminant analysis, SVM, logistic regression, nearest neighbours, and random forest. Ground truth was established based on medical history and vPSG. Features were selected based on correlation with the two groups, followed by least absolute shrinkage and selection operator regularisation. Classifiers were trained on the data from 14 nights of six participants and repeated 100 times to reduce bias. Performance was evaluated based on accuracy, sensitivity, and specificity. SVM achieved the best results with the data from the sleep laboratory and was applied in the home environment. The model achieved 93.6% accuracy, 100.0% sensitivity, and 89.7% specificity over a 2-week window at home, across all participants [30].

## 4. Discussion

This review identified works that evaluated sleep in PD patients using wearable sensors in unstructured environments. Of the identified studies, the majority had a clinical focus. However, 38% of the studies still focused on validating new technologies or algorithms. Validation was mostly achieved through comparisons with PSG or correlations with clinical scales. The latter method involves an intrinsic limitation: one of the main goals of using wearable technology is to mitigate patient and clinician bias, which is characteristic of scales and questionnaires. Secondly, wearable devices might detect subclinical outcomes and allow for the analysis of temporal metrics that are not reflected in these scales.

There is an evident lack of standardisation across study methodologies. One of the few common characteristics observed was the widespread use of accelerometers, which were employed in all but one study. Accelerometers are frequently employed, as they are an inexpensive and unobtrusive way of measuring movement. In these studies, they were mostly positioned on the wrist. However, there is no clear agreement on which side to position the sensor. For example, studies that extracted parameters related to bradykinesia and dyskinesia positioned the sensor on the side most affected by disease. Conversely, studies that analysed actigraphic parameters frequently positioned the sensor on the non-dominant side to diminish the interference of hand movement. Similar reasoning was given in studies that placed the sensor on the side least affected by the disease. One study [30] tested the performance of their algorithm on both sides, coming to the conclusion that the most affected side achieved better results at distinguishing between RBD and non-RBD. 32% of studies did not specify the side of sensor placement. The second most common placement of accelerometers was on the chest, where they were primarily used to analyse turning kinematics.

The reviewed studies extracted a broad range of parameters related to sleep. Temporal descriptors of sleep were mainly derived from the parameters extracted via PSG. However, these parameters often had distinct definitions in the different studies. The lack of consistency hindered comparisons across studies. Movement parameters were affected by similar limitations derived from a lack of consensus in their definitions. Additionally, the use of activity counts (expressed in arbitrary units) instead of acceleration further prevents comparison.

While some of the included studies used wearable technology to study specific sleep disorders that affect PD patients, inconsistent results across studies highlight the need for further validation studies and larger cohorts.

With the exception of Africa, all inhabited continents were represented by at least one country of recruitment. As expected, clinically focused studies included more participants than validation studies. There was an underrepresentation of the female sex across all studies, with only 29% of included individuals with PD being female. This is of significant importance, as it has been shown that PD manifests differently between sexes [36]. The age of the included PD patients reflects the age group typically affected by PD. However, the different age ranges between the included PD patients and controls add a confounding variable that needs to be controlled for [37]. Patients with advanced PD were also underrepresented, with only one study including patients with H&Y stage 5. This is again a significant limitation, as sleep quality is associated with disease progression.

The median duration of monitoring across all studies was 3 days. As shown in the work of Raschellà et al. [30], result accuracy might improve when this value is increased to at least one week. Extending the monitoring period to at least one week may enhance result accuracy by capturing more representative data on patients’ daily living patterns and accounting for behavioural variations throughout the week.

A minority of studies utilised machine-learning algorithms. The studies showed promising results in distinguishing between waking and sleep states, individuals with PD and controls, good and poor sleep quality, and RBD versus controls. However, ML warrants further exploration. As is discussed in the research by Espay et al., technology should be explored with the goal of improving current tools, rather than simply finding sensor-based versions of existing clinical scores [38]. Notably, this review did not identify studies that applied unsupervised learning or convolutional neural networks, both of which present opportunities for defining new scoring metrics and uncovering novel data patterns.

Based on the quality assessment, future studies should ensure that recruited patients and controls are representative of the population they were recruited from. The demographics and clinical history of participants should be extensively described to ensure that data are comprehensive and comparable. Algorithm validation should not be limited to the association of results with clinical scores. Rather, new metrics should be concomitantly developed to characterise patients. The use of accelerometers should be explored to best define a standardised protocol for this specific population. However, as sensing technology evolves and becomes less cumbersome, other sensing modalities should be integrated into ambulatory monitoring to provide a more in-depth assessment of patients. For example, PPG and electrography can be added to directly measure various physiological signals that complement the information acquired from the accelerometers. The supplementary information can then be explored through machine learning methods suited to multidimensional databases.

While this review has managed to summarise how wearable technology has been used to study sleep in PD, it must be noted that the search strategy was not completely successful at identifying all papers covering this topic. For example, the study by Louter et al. [39] meets all inclusion criteria but was not identified by the search queries.

## 5. Conclusions

This review has provided a comprehensive summary of how wearable technology is currently being employed to study sleep quality and sleep disorders in Parkinson’s disease patients in free-living environments. Across all studies, metrics related to movement, sleep duration, and physiological parameters were extracted to compare PD patients with controls and detect specific sleep disorders such as RBD and nocturnal hypokinesia. Studies are often currently hampered by small sample sizes, variable methodologies, and variable metric definitions. To enhance the understanding of sleep disturbances in PD, future research should prioritise the development of standardised protocols that facilitate cross-study comparisons and improve the reliability of findings.

## Figures and Tables

**Table 1:** Example of search query for the Pubmed database.

Topic	Pubmed Query
Wearable sensors	(“Wearable Electronic Devices”[MeSH] or “Actigraphy”[MeSH] or “Accelerometry”[MeSH] or Wearable* or body-worn or accelerometer* or gyro* or actigraph* or smartphone* or remote sensing or smartwatch* or inertial measurement unit* or IMU or IMUs or mobile device* or sensor*)
	AND
Parkinson’s disease	(“Parkinson Disease”[MeSH] or Parkinson’s disease or Parkinson’s or Parkinson or Parkinson disease)
	AND
Sleep	(“Sleep Wake Disorders”[MeSH] or sleep[MeSH] or sleep or insomnia or dyssomnia or REM or RBD or restless legs or EDS or nocturnal)
	AND
Ambulatory monitoring	(“Monitoring, Ambulatory”[MeSH] or ambulatory or home or free living or homebased or ecological* or continuous or unsupervised or remote)

**Table 2:** Main characteristics of the included studies.

Study	Year	Main Goal	Key Takeaways	Sensing Modalities	Accelerometer Position	Wrist Side	Monitoring Duration
[9]	2019	Validate an ambulatory monitoring device for the detection of sleep and wake states using PSG and study sleep quality in patients with PD.	There was no significant difference between sleep parameters detected via the device and PSG. PD is associated with lower distal skin temperature, sleep efficiency and sleep time and greater WASO, activity during sleep and duration of naps, and a worse circadian function index.	TemperatureAccelerometerLightWrist position	Wrist	LA	7
[10]	2019	Investigate temporal associations between objective and subjective sleep measures and daytime functioning using smartphone-based questionnaires and actigraphy.	Objective sleep did not predict any daytime variables. Subjective sleep quality was predicted via positive affect, but objective sleep quantity and quality were not.	Accelerometer	Wrist	NS	14
[11]	2017	Assess the severity of nocturnal hypokinesia and sleep positions in PD patients and their spouses.	PD patients had significantly fewer episodes of turns in bed, slower turning speed and acceleration, and turned fewer degrees than their spouses. These differences were more significant in the second half of the night. PD patients spent more time in a supine position than their spouses.	AccelerometerGyroscope	WristAnkleChest	Both	1
[12]	2020	Study the prevalence of sleep disturbances and nocturnal hypokinesia in different stages of PD and their relation to nonmotor symptoms and medication dose.	Patients with advanced PD had more upright periods, fewer turns, and a slower turning velocity. Turning duration, degree, and velocity were already altered in recently diagnosed patients compared to controls. Reduced nocturnal movements were associated with PD motor severity, dysautonomia, cognition, and dopaminergic medication.	Accelerometer	Lumbar	NA	2
[13]	2013	Assess the prevalence of sleep disturbance in newly diagnosed PD patients using questionnaires, respiratory home monitoring, and actigraphy.	Subjective sleep measures were not associated with objective sleep-disordered breathing or periodic limb movements of sleep (PLMS). PLMS was associated with PD severity.	Accelerometer	Foot	NA	3
[14]	2018	Test a wrist-worn device with machine-learning processing to assess PD patients based on circadian rhythm, motor, and autonomic disruption.	The device reliably collected reliable information about motor (acceleration and time in movement) and non-motor (sleep and skin temperature rhythms) features. Acceleration during the daytime, time in movement during sleep, and their ratio (A/T) were the best indexes to characterise PD symptoms.	TemperatureAccelerometerLightWrist position	Wrist	LA	7
[15]	2023	Validate a new wearable system, composed of an array of dry electrodes, to accurately measure sleep at home using PSG.	The average total agreement between sleep stage classification via the two systems was 77.25%. Agreement did not vary between the PD and the control group. The system detected RSWA with a sensitivity of 85.7%. WASO was significantly higher when measured in the sleep lab when compared with the at-home recording.	EEGEOGEMG	NA	NA	1
[16]	2017	Develop an objective assessment of a patient’s ability to turn in bed in their own home environment.	The number, degree, velocity, acceleration, and torque of axial rotations of PD patients in bed were significantly less than those of their spouses. Significant correlations were observed between the torque of turning in bed and UPDRS and total nocturnal akinesia dystonia and cramp scores.	AccelerometerGyroscope	Chest	NA	2
[17]	2015	Identify the wake/sleep status in PD patients for closed-loop deep brain stimulation.	The wake/sleep state identification for the chest algorithm, when compared with video recordings, achieved an accuracy of 85.78% and 82.74%, respectively, for patients with DBS on and DBS off. The algorithm performance for the chest was comparable to that of the commonly used location on the wrist.	Accelerometer	WristChest	ND	1
[18]	2022	Develop a tool for an automated diagnosis of the prodromal state of Parkinsonian syndromes based on sleep actigraphy.	The sleep/wake classifier achieved an accuracy of 83%. The developed diagnosis method distinguishes participants with prodromal Parkinsonian syndromes and healthy controls with 94% accuracy, 100% sensitivity, and 91% specificity.	TemperatureLightAccelerometer	Wrist	NS	6 ^†^
[19]	2014	Study the interdaily stability (IS) and intradaily variability to describe rest–activity rhythm using simulated and actigraphic data.	Rhythmic synchronisation of activity and rest was significantly higher in young adults than adults with PD when the average IS was considered; however, this difference was not seen when using the IS calculated with a sample frequency of 60 min. PD patients showed reduced activity compared to young individuals.	Accelerometer	Wrist	NS	7
[20]	2016	Employ multiple inertial measurement units to compare nocturnal movements between PD patients and their spouses and correlate these metrics with disease severity scores.	PD patients had fewer turning movements and turned with a lower degree, velocity, and acceleration than their spouses. However, PD patients had more episodes of getting out of bed. Nocturnal hypokinesia was correlated with daytime axial and nonmotor symptoms. Leg movements were correlated with clinical scores. Nocturia was correlated with medication dose.	AccelerometerGyroscope	WristAnkleChest	Both	1
[21]	2024	Extract metrics that characterise nighttime motility and develop a method for the automatic assessment of sleep quality.	SVM was the best-performing classifier (96.2% accuracy) at distinguishing between HC and PD. XGBoost achieved 85.7% accuracy at differentiating between good and bad sleep quality.	AccelerometerGyroscopeMagnetometer	Chest	NA	1
[22]	2023	Analyse sleep architecture in PD patients and correlate sleep data with head position and motor and non-motor symptoms.	Sleep architecture was consistent across nights. Sleep was predominantly performed in the supine position. REM sleep in the supine decubitus was associated with disease duration and motor symptoms. No correlation was found between sleeping position and medication dose.	AccelerometerEEGEOGEMGMicrophonePPG	Head	NA	3
[23]	2024	Analyse the correlation between clinical scores of non-motor symptoms and passively collected digital data related to activity, sleep, and vital signs.	Digital measures of sleep correlated with clinical measures of cognition, autonomic function, and medication but did not correlate with psychiatric or motor clinical measures. Digital data could not predict scores of questionnaires or scales using linear regression. Digital outcome measures were significantly better at detecting change than clinical ones.	AccelerometerGyroscopePPGECG	Wrist	NS	6
[24]	2013	Evaluate the time–course of the sleepiness level during the wakefulness period in untreated patients with early-stage Parkinson’s disease.	A higher level of sleepiness was found in the patients than the controls in the hours following awakening and in the early afternoon.	Accelerometer	Wrist	NS	3
[25]	2024	Compare daily light exposure between patients with PD and non-PD older adults and evaluate the association of daily light exposure with objective sleep measures in patients with PD.	Greater daytime light exposure and lower nighttime light exposure were significantly associated with better objective sleep measures in patients with PD.	AccelerometerLight	Wrist	ND hand	7
[26]	2018	Evaluate the accuracy of the NIMBLE wearable biosensor patch to record body movements in clinic and home environments versus clinical measurement of motor symptoms.	No discernable relationship was identified between the total amounts of motor activity, or total time lying down during sleep and the quality of the sleep pattern descriptors reported by participants via a diary app.	AccelerometerEMG	ChestShinForearmHand	MA	3
[27]	2011	Evaluate the alteration of circadian rhythm in PD patients, by investigating rest activities and autonomic function.	PD patients have lower activity levels when out of bed and higher activity levels when in bed, and the circadian rest–activity rhythm in PD decreases with disease severity. HRV showed that the total frequency component and low-frequency/high-frequency ratio were low in PD patients, suggesting that autonomic activities and the circadian rhythm of the sympathetic nervous system are attenuated in PD.	AccelerometerECG	Wrist	LA	7
[28]	2008	Compare rest–activity rhythms in healthy older adults and PD patients with and without hallucinations.	PD patients demonstrated a reduced amplitude of activity and increased intradaily variability compared to healthy older adults, independently of age and cognitive status. Hallucinators showed lower interdaily stability, significantly greater activity during the ‘‘nighttime’’, and a significantly reduced relative amplitude of activity compared to nonhallucinators, independently of clinical factors including motor fluctuations.	Accelerometer	Wrist	LA	7
[29]	2016	Develop an inertial sensor that can provide quantitative monitoring of axial rotation of patients with PD and their spouses while in bed.	Patients with PD rolled over significantly fewer times than their spouses, and the position change was significantly smaller in patients with PD. Patients with PD rolled over at a significantly slower speed and acceleration than their spouses. In contrast, patients with PD got out of bed significantly more often than their spouses. It is technically feasible to develop an easy-to-use, portable, and accurate device that can assess nocturnal movements of patients with PD.	AccelerometerGyroscope	Chest	NA	1
[30]	2023	Explore the use of wrist actigraphy to enable automatic RBD diagnoses in home settings.	SVM achieved the best performance in distinguishing between RBD and non-RBD patients, with an accuracy of 92.9% for in-lab data. Maximum performance was achieved with the actigraph on the wrist of the most affected side. Over 7 days of at-home data, the classifier achieved 100% accuracy for PD patients.	AccelerometerLight	Wrist	MA	14
[31]	2014	Study the relation between daytime immobility and sleepiness using actigraphy.	There was concordance between immobility and PSG scores in 85.6% epochs. PD patients with high ESS had significantly higher PTI than other participants. PD patients with a high PTI had more bradykinesia, less dyskinesia, and higher PDQ39 scores than those with low PTI. There was no relationship between PTI and dose or type of PD medications. PTI increased in the 30–60 min after levodopa.	Accelerometer	Wrist	MA	10
[32]	2017	Explore the feasibility of using wearable devices to quantitatively measure the daily activity in patients with Parkinson’s disease (PD) and to monitor medication-induced motor fluctuations.	Daily sleep time was significantly lower in PD patients than in the control group.	Accelerometer	Wrist	NS	3
[33]	2020	Investigate the relationship between daytime sleepiness and other non-motor and motor fluctuations in people with PD.	Episodes of daytime sleepiness, as reported by home diaries, were associated with other self-reported non-motor and motor fluctuations but were not supported with PKG data.	Accelerometer	Wrist	MA	6
[34]	2008	Find out how the daytime and night-time motor activity levels in individuals without motor disorders differ from those of patients with Parkinson’s disease.	PD patients had 1.5–2-fold lower daytime motor activity but also showed 1.5–2-fold higher motor activity at nighttime. Older controls showed a lower daytime but similar nighttime motor activity when compared to younger controls. A ratio of nighttime to daytime motor activity could clearly distinguish controls and patients.	Accelerometer	Wrist	ND	3

LA: least affected; MA: most affected; ND: non-dominant; NS: not specified; NA: not applicable; ^†^: estimated.

**Table 3:** Quality assessment based on the checklist developed by Downs and Black [7].

	Reporting								External Validity		Internal Validity—Bias				Internal Validity—Confounding				
Study	1	2	3	5	6	7	9	10	11	12	16	17	18	20	21	22	25	26	Total (%)
[9]	1	1	1	2	1	1	NA	1	0	0	1	NA	1	1	0	0	1	NA	73%
[10]	1	0	1	NA	1	1	NA	1	0	0	1	NA	1	1	0	NA	0	NA	62%
[11]	1	1	1	2	1	1	NA	1	0	0	1	NA	1	1	1	0	1	NA	81%
[12]	1	1	1	2	1	1	NA	1	0	0	1	NA	1	1	0	0	1	NA	75%
[13]	1	1	1	1	1	1	NA	0	1	0	1	NA	1	1	1	0	0	NA	69%
[14]	1	1	1	2	0	1	NA	0	0	0	1	NA	1	0	0	0	1	NA	56%
[15]	1	1	1	NA	1	1	NA	1	0	0	1	NA	1	1	0	0	1	NA	71%
[16]	1	1	1	2	1	1	NA	1	0	0	1	NA	1	1	1	0	1	NA	81%
[17]	1	1	0	0	1	1	NA	NA	0	0	1	NA	1	1	0	0	0	NA	47%
[18]	1	0	0	0	1	1	NA	NA	0	0	1	NA	1	1	NA	NA	0	NA	46%
[19]	1	0	0	0	1	1	NA	1	0	0	1	NA	1	1	0	0	0	NA	44%
[20]	1	1	1	2	1	1	NA	1	0	0	1	NA	1	1	1	1	1	NA	88%
[21]	1	1	0	2	1	1	NA	0	0	0	1	NA	1	1	0	0	0	NA	56%
[22]	1	1	1	NA	1	1	NA	1	0	0	1	NA	1	1	NA	NA	NA	NA	82%
[23]	1	0	0	NA	1	1	0	1	0	0	1	1	1	1	NA	NA	NA	0	57%
[24]	1	1	1	2	1	1	NA	0	1	0	1	NA	1	1	0	0	1	NA	75%
[25]	1	1	1	2	1	1	NA	1	0	0	1	NA	1	1	0	0	0	NA	69%
[26]	1	0	1	NA	1	1	NA	0	0	0	1	NA	1	1	NA	NA	NA	NA	64%
[27]	1	0	1	2	1	1	NA	1	1	0	1	NA	1	1	1	1	1	NA	88%
[28]	1	1	1	2	1	1	NA	0	0	0	1	NA	1	1	0	0	1	NA	69%
[29]	1	1	1	2	1	1	NA	1	0	0	1	NA	1	1	1	1	0	NA	81%
[30]	1	1	1	2	1	1	NA	1	1	0	1	NA	1	1	0	0	1	NA	81%
[31]	0	0	0	1	1	1	NA	1	0	0	1	NA	1	1	1	0	1	NA	56%
[32]	1	0	1	2	1	1	NA	1	0	0	1	NA	1	1	0	0	1	NA	69%
[33]	1	1	1	2	1	1	NA	0	0	0	1	NA	1	1	1	1	1	NA	81%
[34]	1	1	1	2	1	1	NA	0	0	0	1	NA	1	1	1	0	1	NA	75%
**Total (%)**	96%	69%	77%	81%	96%	100%	0%	67%	15%	0%	100%	100%	100%	96%	41%	19%	65%	0%	

NA: Not applicable.

**Table 4:** Demographics of included participants.

Study	No. of PDP	No. of Controls	Age of PDP	Age of Controls	PDP% Female	Control% Female	MinH&Y	MaxH&Y	Countries
[9]	15	HC: n = 15Sleep Disorders: n = 70	65.53 ± 2.19 ^†^ y	60.71 ± 1.97 ^†^ y	20	20	NS	NS	Spain
[10]	20	0	66.5 ± 9.3 y	NA	35.0	NA	1	4	USA
[11]	18	18	64.9 ± 7.6 y	63.8 ± 8.5 y	22.2	77.8	NS	NS	Thailand
[12]	304	205	68.4 ± 8.5 y	66.3 ± 13.3 y	35	66	1	3	Belgium, Israel, Italy, Netherlands, UK
[13]	106	99	66.5 ^‡^(60.1–74.1) y	67.9 ^‡^ y	36.8	45.5	1	3	UK
[14]	12	12	65.8 ± 2.67 ^†^ y	69.41 ± 1.90 ^†^ y	25.0	25.0	2	3	Spain
[15]	29	21	65.4 ± 7.6 y	56.6 ± 8.4 y	34.5	38.1	1	3	NS
[16]	17	17	64.9 ± 7.9 y	64.3 ± 8.6 y	29.4	70.6	NS	NS	Thailand
[17]	12	13	64.5 ± 2.0 y	Young HC: 24.8 ± 1.53 y Old HC: 61.5 ± 0.5 y	NS	NS	NS	NS	China
[18]	NS	HC, and participants with dementia with Lewy bodies and mild cognitive impairment	NS	NS	NS	NS	NS	NS	NS
[19]	26	24	(38–69) y	(18–23) y	NS	NS	NS	NS	Brazil
[20]	19	19	64.63 ± 7.95 y	64.32 ± 8.46 y	26.3	73.7	1,5	3	Thailand
[21]	12	28	68 ± 4.1 y	38 ± 10.7 y	35.7	35.7	NS	NS	Italy
[22]	20	0	65.7 ± 8.6 y	NA	50	NA	1	3	Italy
[23]	149	0	67.69 ± 7.54 y	NA	NS	NA	NS	NS	NS
[24]	18	18	68.39 ± 1.89 ^†^ y	67.22 ± 1.98 ^†^ y	50.0	50.0	1	2	Italy
[25]	189	1101	71.3 ± 7.6 y	71.9 ± 7.1 y	46.6	53.2	1	5	Japan
[26]	21	0	65 ± 7 y	NA	42.9	NA	2	3	USA
[27]	27	30	69.33 ± 7.29 y	68.93 ± 5.12 y	37	47	1	4	Japan
[28]	50	29	73.36 ± 7.54 y	70.90 ± 5.59 y	24	31	NS	NS	UK
[29]	6	6	65.5 ± 7.45 y	66.67 ± 7.76 y	0	100	NS	NS	Thailand
[30]	26	Insomnia: n = 18	68.02 ± 10.6 y	52.7 ± 15.3 y	26.9	33.3	1.5	3	Switzerland
[31]	68	30	65.9 ^‡^(40–80) y	65.8 ^‡^ y	NS	NS	NS	NS	Australia
[32]	21	20	66.52 ± 9.13 y	63.15 ± 8.70 y	23.8	25.0	1	4	China
[33]	52	0	65.3 ± 10.5 y	NA	38.5	NA	1	4	Sweden
[34]	17	69	74.88 ± 7.2 y	53.47 ± 21.03 y	47.1	65.2	1	3	Germany

Age: mean ± std; ^†^: standard error; ^‡^: median; NA: not applicable; NS: not specified; HC: healthy control; PDP: PD patient.

**Table 5:** Sleep disorders in Parkinson’s disease.

Sleep Disorder	Studies	Main Outcomes
Nocturnal hypokinesia	[11,12,16,20,29]	Number, degree, velocity, acceleration, and torque of turns is reduced in PD patients. The difference increases throughout the night and with disease severity.
Nocturia	[11,20,29]	Getting out of bed was more frequent in PD patients than controls and was associated with nocturia.
Restless legs syndrome	[13]	Periodic limb movements were not associated with symptomatic restless legs syndrome.
RBD	[15,23,30]	An electrode array positioned in the head allowed the detection of RBD with 92% accuracy. An RBD classifier based on actigraphic features achieved 100% accuracy.
Extreme daytime sleepiness	[23,31,33]	Periods of immobility measured via actigraphy were associated with extreme daytime sleepiness when measured with the ESS but not the KSS.

## Data Availability

No new data were created or analysed in this study. Data sharing is not applicable to this article.

## Supplementary Materials

The following supporting information can be downloaded at: https://www.mdpi.com/article/10.3390/bios15040212/s1, Table S1: List of wearable sensors used in each study.

## Abbreviations

The following abbreviations are used in this manuscript:

ACM	Ambulatory circadian monitoring
DBS	Deep brain stimulation
ECG	Electrocardiography
EDS	Excessive daytime sleepiness
EEG	Electroencephalography
EMG	Electromyography
EOG	Electrooculography
ESS	Epworth Sleepiness Scale
H&Y	Hoehn and Yahr Scale
HCs	Healthy controls
HRV	Heart rate variability
IS	Interdaily stability
IV	Intradaily variability
MeSH	Medical Subject Headings
ML	Machine learning
MMSE	Mini-Mental State Examination
MoCA	Montreal Cognitive Assessment
PD	Parkinson’s disease
PDQ39	The Parkinson’s Disease Questionnaire
PLMI	Periodic limb movements
PPG	Photoplethysmography
PRISMA	Preferred Reporting Items for Systematic Reviews and Meta-Analyses
PSG	Polysomnography
PSQI	Pittsburgh Sleep Quality Index
PTI	Proportion of time immobile
RBD	REM sleep behaviour disorder
RBDSQ	RBD screening questionnaire
REM	Rapid eye movement
RLS	Restless legs syndrome
RSWA	REM sleep without atonia
SE	Sleep efficiency
SOL	Sleep onset latency
SVM	Support vector machine
SWA	Slow wave activity
TST	Total sleep time
UPDRS	Unified Parkinson’s disease rating scale
vPSG	Video polysomnography
WASO	Wake after sleep onset
